# Activation of IP_3_ receptors requires an endogenous 1-8-14 calmodulin-binding motif

**DOI:** 10.1042/BJ20121034

**Published:** 2012-12-07

**Authors:** Yi Sun, Ana M. Rossi, Taufiq Rahman, Colin W. Taylor

**Affiliations:** Department of Pharmacology, University of Cambridge, Cambridge CB2 1PD, U.K.

**Keywords:** 1-8-14 motif, calcium signalling, calmodulin, inositol 1,4,5-trisphosphate receptor, myosin light chain kinase (MLCK), BCR, B-cell receptor, CaBP1, Ca^2+^-binding protein 1, CaM, calmodulin, CLM, cytosol-like medium, IP_3_, inositol 1,4,5-trisphosphate, IBC, IP_3_-binding core, IP_3_R, IP_3_ receptor, MLCK, myosin light chain kinase, NT, N-terminus, RyR, ryanodine receptor, SD, suppressor domain

## Abstract

Binding of IP_3_ (inositol 1,4,5-trisphosphate) to the IP_3_-binding core (residues 224–604) of IP_3_Rs (IP_3_ receptors) initiates opening of these ubiquitous intracellular Ca^2+^ channels. The mechanisms are unresolved, but require conformational changes to pass through the suppressor domain (residues 1–223). A calmodulin-binding peptide derived from myosin light chain kinase uncouples these events. We identified a similar conserved 1-8-14 calmodulin-binding motif within the suppressor domain of IP_3_R1 and, using peptides and mutagenesis, we demonstrate that it is essential for IP_3_R activation, whether assessed by IP_3_-evoked Ca^2+^ release or patch-clamp recoding of nuclear IP_3_R. Mimetic peptides specifically inhibit activation of IP_3_R by uncoupling the IP_3_-binding core from the suppressor domain. Mutations of key hydrophobic residues within the endogenous 1-8-14 motif mimic the peptides. Our results show that an endogenous 1-8-14 motif mediates conformational changes that are essential for IP_3_R activation. The inhibitory effects of calmodulin and related proteins may result from disruption of this essential interaction.

## INTRODUCTION

Ca^2+^ channels allow most electrical and many chemical signals to be transduced into the changes in cytosolic Ca^2+^ concentration that regulate almost every aspect of cellular activity [1]. Most Ca^2+^ channels are also regulated by Ca^2+^, either directly or via CaM (calmodulin) [2]. This provides feedback regulation of Ca^2+^ signalling and it allows Ca^2+^ channels to evoke regenerative Ca^2+^ signals [3]. The latter are important because they underpin the versatility of Ca^2+^ as an intracellular messenger, permitting it to function either locally or globally [1].

Two major families of intracellular Ca^2+^ channels, IP_3_Rs [IP_3_ (inositol 1,4,5-trisphosphate) receptors] and RyRs (ryanodine receptors), share many structural [4,5] and functional [5–7] properties. Most notably, all IP_3_Rs and RyRs are stimulated by low concentrations of cytosolic Ca^2+^ and inhibited by higher concentrations. Ca^2+^-binding sites within the RyR itself can mediate this biphasic Ca^2+^ regulation [7], but, for IP_3_Rs, it remains unclear whether additional Ca^2+^-binding proteins are required [6]. None of the many Ca^2+^-binding sites in RyRs [8] or IP_3_Rs [9] has been unambiguously associated with Ca^2+^ regulation of channel gating [10,11], although mutation of a single equivalent residue in RyRs or IP_3_Rs (Glu^2100^ in IP_3_R1) modulates their Ca^2+^-sensitivity [11]. Both families of intracellular Ca^2+^ channels are also regulated by CaM, a ubiquitously expressed and highly conserved Ca^2+^-binding protein [12]. Related proteins with EF-hand Ca^2+^-binding structures, such as S100A and CaBP1 (Ca^2+^-binding protein 1), also regulate RyRs and IP_3_Rs, but the physiological significance of these interactions between intracellular Ca^2+^ channels and CaM or related proteins is unresolved [13,14]. Despite some conflicting evidence [15], CaM seems not to be essential for Ca^2+^ regulation of RyRs or IP_3_Rs [16–18], but it does regulate both channels and it modulates their responses to Ca^2+^ [19–21].

**Figure 1:**
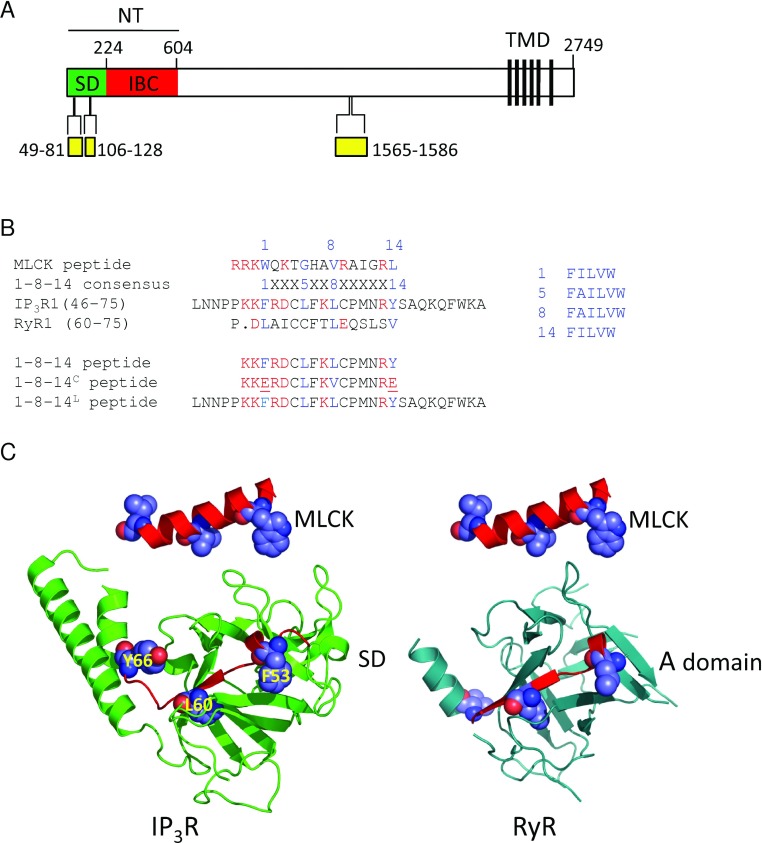
A putative 1-8-14 motif within the SD of the IP_3_R (**A**) Key features of a rat IP_3_R showing the NT, with its component parts (SD and IBC), the six C-terminal transmembrane domains (TMD) that form the pore and the CaM-binding domains (yellow). Residue numbers are shown. (**B**) Comparison of 1-8-14 motifs showing the conserved hydrophobic residues of the consensus sequence in blue. Charged residues within the 1-8-14 motif are highlighted in red because the consensus motif has a net charge of +3 to +6. The lower panel shows the peptides used with mutated residues underlined. (**C**) Structure of the SD of IP_3_R1 (PDB code 1XZZ) and the equivalent region (A domain) of RyR1 (PDB code 3HSM) with the pseudo-1-8-14 motif highlighted and compared with MLCK in the structure it adopts when bound to Ca^2+^–CaM (PDB code 1QTX).

All IP_3_Rs are inhibited by Ca^2+^–CaM [22], but neither of the two CaM-binding sites within IP_3_R1, nor a third that is created by alternative splicing [23], clearly mediates this inhibition of IP_3_-evoked Ca^2+^ release. The central site [24] (Figure 1A) mediates neither Ca^2+^ nor CaM regulation of IP_3_R activity [16,17] and it is absent from IP_3_R3. The functional role of the split N-terminal site (Figure 1A), one component of which may also bind CaBP1 [25], is also unclear. It has been proposed to bind CaM and thereby to inhibit IP_3_R activity, but only when Ca^2+^ has bound elsewhere [26]. The evidence that CaM inhibits IP_3_R only in the presence of Ca^2+^, without CaM itself providing the Ca^2+^-sensor, is persuasive [26], but there is no compelling evidence to link this to the N-terminal CaM-binding site [27].

The links between CaM binding and function are better understood for RyRs, although the effects differ between RyR subtypes [7]. A single site on each RyR1 subunit (residues 3614–3643 in rabbit RyR1), which is conserved in all RyRs, binds the C-terminal lobe of both apo-CaM and Ca^2+^–CaM and appears to mediate the functional effects of CaM [20,28,29]. As this tethered CaM binds Ca^2+^, it migrates towards the NT (N-terminus) of the binding site and the CaM switches from activating RyR1 to inhibiting it [19]. The CaM-binding site of RyR1 also engages other CaM-like domains, notably the C-terminus of the L-type Ca^2+^ channel which inhibits RyR1 activity [30], and perhaps an EF-hand-like structure within the C-terminal region of RyR1 which binds Ca^2+^ and modulates Ca^2+^ regulation of RyR [31]. These observations suggest that the CaM-binding domain of RyR also mediates important inter- and intra-molecular interactions, and that the complex effects of CaM and related proteins may, at least in part, result from disrupting these interactions [29,31,32].

For IP_3_Rs, IP_3_ binding to the IBC (IP_3_-binding core) (residues 224–604) (Figure 1A) initiates the conformational changes that lead to opening of a pore formed by the C-terminal transmembrane domains of each of the four IP_3_R subunits [5,33]. These conformational changes pass via the N-terminal SD (suppressor domain) (residues 1–223), which is essential for IP_3_R activation. Indeed, the major conformational changes associated with IP_3_R activation appear to occur within the NT (residues 1–604) [5,33]. Although both IP_3_ and Ca^2+^ are required for IP_3_R activation [6,34], it is not yet clear how the conformational changes initiated by IP_3_ lead to Ca^2+^ binding and then to gating of the pore. It is therefore intriguing that a CaM-binding peptide derived from MLCK (myosin light chain kinase), which comprises a 1-8-14 CaM-binding sequence [35], reversibly inhibits IP_3_-evoked Ca^2+^ release [36] via all three vertebrate IP_3_R subtypes. Furthermore, MLCK peptide is more potent in the presence of Ca^2+^ [35]. This inhibition is entirely independent of CaM and involves interaction of MLCK peptide with the NT in a manner that requires the SD [35]. We speculate, by analogy with RyRs, that inhibition of IP_3_Rs by MLCK peptide might result from disruption of an interaction between endogenous CaM-like and CaM-binding domains within IP_3_Rs, and that, for IP_3_Rs, this interaction is essential for activation. In the present study, we explored this hypothesis further.

## EXPERIMENTAL

### Materials

Cell culture materials were from Gibco, except for fetal bovine serum (Sigma). CaM purified from bovine brain was from Calbiochem. [^3^H]IP_3_ (18 Ci/mmol) was from PerkinElmer. IP_3_ was from Alexis Biochemicals. Peptides were synthesized and purified by Sigma or New England Peptide, and each was shown to be >90% pure by HPLC. The peptide sequences are listed in Supplementary Table S1 (at http://www.BiochemJ.org/bj/449/bj4490039add.htm).

### Site-directed mutagenesis

The NT (residues 1–604) and IBC (residues 224–604) of rat IP_3_R1 were amplified by PCR from the full-length receptor clone lacking the SI splice region (GenBank® accession number GQ233032.1) as described previously [33]. The fragments were ligated into pTrcHis A (Invitrogen) to allow expression of N-terminally His_6_-tagged proteins. Mutagenesis of the 1-8-14 motif within the NT used the QuikChange® II XL site-directed mutagenesis kit (Stratagene) for single mutants (F53E, L60E, Y66E and K52E) and the QuikChange® multi-site-directed mutagenesis kit for the double mutant (F53E and Y66E). The primers used are listed in Supplementary Table S2 (at http://www.BiochemJ.org/bj/449/bj4490039add.htm). The same primers and conditions were used for mutagenesis of full-length IP_3_R using IP_3_R1 in the pENTR 1A vector. Full-length constructs were subcloned into pcDNA3.2/V5-DEST for expression in DT40 cells. The complete sequence of every mutant construct was verified by sequencing.

### Culture and stable transfection of DT40 cells

DT40 cells in which the genes for all three IP_3_R subtypes had been disrupted (DT40-KO) [37] and DT40 cells stably expressing rat IP_3_R1 (DT40-IP_3_R1) were grown in RPMI 1640 medium supplemented with 10% (v/v) fetal bovine serum, 1% (v/v) heat-inactivated chicken serum, 2 mM l-glutamine and 50 μM 2-mercaptoethanol. Cells were grown in suspension in 175 cm^2^ flasks at 37°C in an atmosphere of 5% CO_2_. They were used or passaged when they reached a density of ~2×10^6^ cells/ml. To generate stable cell lines expressing mutant IP_3_R, the mutant construct in pcDNA3.2/V5-DEST was linearized, and DT40 cells were transfected by nucleofection (Amaxa, protocol B-23). Cell lines were selected with G-418 (2 mg/ml) and screened initially by Western blotting using a peptide antiserum to IP_3_R1 [38] as described previously [33], and then using the functional assay described below.

### Ca^2+^ release from the intracellular stores of permeabilized cells

The free Ca^2+^ concentration of the intracellular stores of permeabilized cells was measured using a low-affinity Ca^2+^ indicator trapped within the endoplasmic reticulum as reported previously [39]. Briefly, DT40 cells (4×10^7^ cells/ml) were suspended in HBS (Hepes-buffered saline: 135 mM NaCl, 5.9 mM KCl, 11.6 mM Hepes, 1.5 mM CaCl_2_, 11.5 mM glucose and 1.2 mM MgCl_2_, pH 7.3) containing 1 mg/ml BSA, 0.4 mg/ml Pluronic F127 and 20 μM mag-fluo-4/AM (Invitrogen). After 1 h at 20°C in the dark with gentle shaking, cells were centrifuged at 650 ***g*** for 2 min and resuspended to 10^7^ cells/ml in Ca^2+^-free CLM (cytosol-like medium) (20 mM NaCl, 140 mM KCl, 1 mM EGTA, 20 mM Pipes and 2 mM MgCl_2_, pH 7.0) containing 20 μg/ml saponin. After incubation at 37°C with gentle shaking for 4 min, permeabilized cells were centrifuged at 650 ***g*** for 2 min and resuspended in Mg^2+^-free CLM, supplemented with CaCl_2_ to give a final free Ca^2+^ concentration of 220 nM. The free Ca^2+^ concentration of CLM was calculated using the MaxChelator program (http://maxchelator.stanford.edu) and then measured using fluo-3 or fura-2. Cells were then washed, resuspended in Mg^2+^-free CLM containing 10 μM FCCP (carbonyl cyanide *p*-trifluoromethoxyphenylhydrazone) to inhibit mitochondria, and distributed into a 96-well plate (10^6^ cells in 50 μl of CLM/well). After centrifugation, fluorescence from the luminal indicator was recorded using a FlexStation II platereader (Molecular Devices) equipped to allow automated additions [39]. In all experiments, the intracellular stores were allowed to load to steady-state with Ca^2+^ after addition of MgATP. IP_3_ was then added with thapsigargin (1 μM, to inhibit Ca^2+^ reuptake). The Ca^2+^ release evoked by IP_3_ is expressed as a fraction of the ATP-dependent Ca^2+^ uptake.

### Patch-clamp recording

Currents were recorded from patches excised from the outer nuclear envelope of DT40 cells expressing recombinant rat IP_3_R1 using symmetrical caesium methanesulfonate (140 mM) as the charge-carrier. The composition of recording solutions and methods of analysis were otherwise as described previously [40].

### Expression of N-terminal fragments of IP_3_R

The pTrcHis constructs were used for expression of N-terminally His_6_-tagged proteins in *Escherichia coli* strain BL21(DE3) cells. Before use for [^3^H]IP_3_ binding, proteins were cleaved from the His_6_ tags using biotinylated thrombin (Novagen) at the engineered thrombin-cleavage site [33]. Complete cleavage was verified by Western blotting using an anti-His_6_ antibody. The proteins were used for [^3^H]IP_3_ binding without further purification [33].

### [^3^H]IP_3_ binding

Equilibrium-competition binding assays were performed at 4°C for 5 min in CLM (500 μl) with a free Ca^2+^ concentration of 220 nM and containing [^3^H]IP_3_ (0.75–1.5 nM), bacterial lysate (10 μg of protein for IBC and 100 μg of protein for NT) or cerebellar membranes (50 μg of protein) and competing ligands. Non-specific binding was defined by addition of 10 μM IP_3_. Bound and free [^3^H]IP_3_ were separated by centrifugation at 20000 ***g*** for 5 min, after addition of poly(ethylene glycol) (15% final concentration) and γ-globulin (0.75 mg) for soluble proteins. Results were analysed by fitting to a Hill equation (using GraphPad Prism) from which the IC_50_ (half-maximal inhibitory concentration) and thereby the *K*_d_ (equilibrium dissociation constant) were calculated [33].

### Western blotting

Cells in Ca^2+^-free CLM containing 2-mercaptoethanol (1 mM) and protease inhibitors were lysed by addition of PopCulture (10%), lysozyme (10 μg/ml), DNAse (5 units/ml) and RNAse (10 μg/ml). The proteins were separated using SDS/PAGE pre-cast mini-gels (Invitrogen) and transferred on to a PVDF membrane using an Iblot dry-transfer apparatus (Invitrogen). The primary antibodies were rabbit anti-His_6_ (1:3000 dilution) (Sigma) and anti-IP_3_R1 (1:1000 dilution) [33]. HRP (horseradish peroxidase)-conjugated anti-rabbit secondary antibodies (1:5000 dilution) (AbCam) and the Super Signal West Pico chemiluminescence reagent (Pierce) were used to detect immunoreactivity. Bands were quantified using GeneTools software (Syngene).

### Statistical analysis

For comparisons of *K*_d_, EC_50_ (half-maximally effective concentration) or IC_50_ values, their negative logarithms (p*K*_d_, pEC_50_ and pIC_50_; means±S.E.M.) were used for statistical analyses. For clarity, some Figures show normalized results, but all statistical analyses were performed on the raw data using paired or unpaired Student's *t* tests. *P*<0.05 was considered significant.

## RESULTS AND DISCUSSION

### Reversible inhibition of IP_3_-evoked Ca^2+^ release by an endogenous 1-8-14 peptide

A sequence within the SD of all known IP_3_Rs (residues 53–66 in rat IP_3_R1; Supplementary Figure S1 at http://www.BiochemJ.org/bj/449/bj4490039add.htm) includes the critical hydrophobic residues of a 1-8-14 CaM-binding motif appropriately oriented within the known structure of the SD [41] (Figures 1B and 1C) and with the required net positive charge [35]. The sequence lies within one of the two regions (residues 49–81; Figure 1A) within the NT reported to bind CaM [42] and CaBP1 [14]. A similar sequence is present within the N-terminal of all RyRs (Supplementary Figure S1). To test our hypothesis that inhibition of IP_3_R by MLCK peptide results from disruption of an essential interaction involving an endogenous 1-8-14 motif, we assessed the effects of a peptide derived from this motif (1-8-14 peptide; Figure 1B and Supplementary Table S1) on IP_3_-evoked Ca^2+^ release.

**Figure 2:**
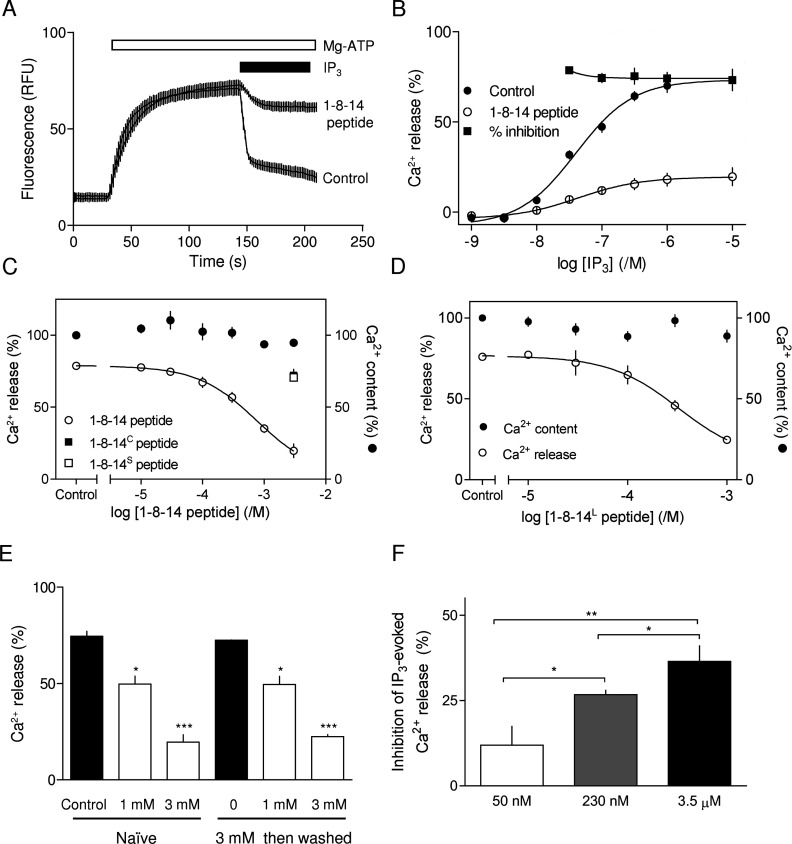
Inhibition of IP_3_R by 1-8-14 peptide (**A**) Typical recording of the free Ca^2+^ concentration within the endoplasmic reticulum of a population of permeabilized DT40-IP_3_R1 cells showing Ca^2+^ uptake after addition of MgATP (1.5 mM), release of Ca^2+^ after addition of IP_3_ (10 μM, with 1 μM thapsigargin to inhibit Ca^2+^ re-uptake) and inhibition of that release by 1-8-14 peptide (3 mM, present throughout as indicated, upper trace). Results are means±S.E.M. for three replicates from a single experiment. (**B**) Concentration-dependent release of intracellular Ca^2+^ stores by IP_3_ alone or after pre-incubation for 2.5 min with 1-8-14 peptide (3 mM). Inhibition by 1-8-14 peptide at each IP_3_ concentration is also shown (%). 1-8-14 peptide caused a significant decrease in the maximal response (*P*<0.001) without significantly changing the sensitivity to IP_3_. (**C** and **D**) Permeabilized cells pre-incubated for 10–20 min with the indicated concentrations of peptide were stimulated with IP_3_ (10 μM, in the continued presence of peptide). Results show the Ca^2+^ content of the stores before addition of IP_3_, and the Ca^2+^ release evoked by IP_3_. (**E**) Permeabilized cells were incubated alone or with 1-8-14 peptide (3 mM) for 10–20 min, washed and then resuspended in CLM. Ca^2+^ release by IP_3_ (10 μM) was then measured after a further incubation for 10–20 min with the indicated concentrations of 1-8-14 peptide. The Ca^2+^ release evoked by IP_3_ with and without peptide is shown for naive cells and after the pre-treatment with 3 mM peptide. The results establish that the effects of 1-8-14 peptide are fully reversible. (**F**) Permeabilized cells pre-incubated with or without 1-8-14 peptide (1 mM) for 10–20 min were stimulated with a maximally effective concentration of IP_3_ in the continued presence of peptide in CLM with the indicated free Ca^2+^ concentration. Results show the inhibition of IP_3_-evoked Ca^2+^ release (%) by 1-8-14 peptide at each free Ca^2+^ concentration. Results in (**B**)–(**F**) are means±S.E.M. (*n*≥3). *P<0.05, **P<0.01 and ***P<0.001.

The 1-8-14 peptide inhibited IP_3_-evoked Ca^2+^ release via IP_3_R1 without affecting either Ca^2+^ uptake or the sensitivity (EC_50_) to IP_3_ (Figures 2A–2D). A maximally effective concentration of the peptide reduced the maximal response to IP_3_ by 77±7%. The IC_50_ for 1-8-14 peptide was 767 μM (pIC_50_, 3.1±0.25) (Figure 2C). Neither a mutant 1-8-14 peptide, in which two critical hydrophobic residues are mutated (1-8-14^C^, 3 mM) nor a scrambled peptide (1-8-14^S^, 3 mM) had any effect on IP_3_-evoked Ca^2+^ release (Figure 2C). Both MLCK peptide (isoelectric point, pI 14.0) and 1-8-14 peptide (pI 11.6) are very basic and might therefore have inhibited IP_3_-evoked Ca^2+^ release by binding directly to IP_3_. We demonstrated previously that this was not the case for MLCK peptide [35], and it is also unlikely for the 1-8-14 peptide. The 1-8-14 and 1-8-14^S^ peptides are equally basic, but only the former inhibited IP_3_R; the percentage inhibition caused by 3 mM 1-8-14 peptide is similar for all IP_3_ concentrations (~75%), and neither was the inhibition reduced by increasing the IP_3_ concentration beyond that required to stimulate maximal Ca^2+^ release (Figure 2B). We conclude that 1-8-14 peptide inhibits IP_3_-evoked Ca^2+^ release by binding to IP_3_R.

The 1-8-14 peptide is only 16 residues long. A longer peptide (30 residues, 1-8-14^L^), which includes additional N- and C-terminal residues that are conserved in all IP_3_Rs (Figure 1B and Supplementary Figure S1), also inhibited IP_3_-evoked Ca^2+^ release without affecting Ca^2+^ uptake (Figure 2D). Although IP_3_R may be slightly more sensitive to the longer peptide (IC_50_, 326 μM; pIC_50_, 3.5±0.25) than to the 1-8-14 peptide (767 μM, 3.1±0.25); the difference was not statistically significant. Subsequent studies used the shorter 1-8-14 peptide because it was less expensive.

The results shown in Figure 2(E) demonstrate that the effects of a maximally effective concentration of 1-8-14 peptide (3 mM) are fully reversible. These experiments, which require extensive washing of the cells between successive challenges with the peptide, confirm that the inhibition of IP_3_Rs by the 1-8-14 peptide, like that by MLCK peptide [35], does not result from dissociation of CaM from IP_3_R [36]. Our previous study demonstrated that MLCK peptide more potently inhibited IP_3_R when the cytosolic free Ca^2+^ concentration was increased [35]. Similar results were obtained with 1-8-14 peptide (Figure 2F). We conclude that 1-8-14 peptide inhibits IP_3_-evoked Ca^2+^ release by binding to the IP_3_R and the inhibition is enhanced at elevated cytosolic Ca^2+^ concentrations.

### Inhibition of single-channel currents through IP_3_Rs by 1-8-14 peptide

**Figure 3:**
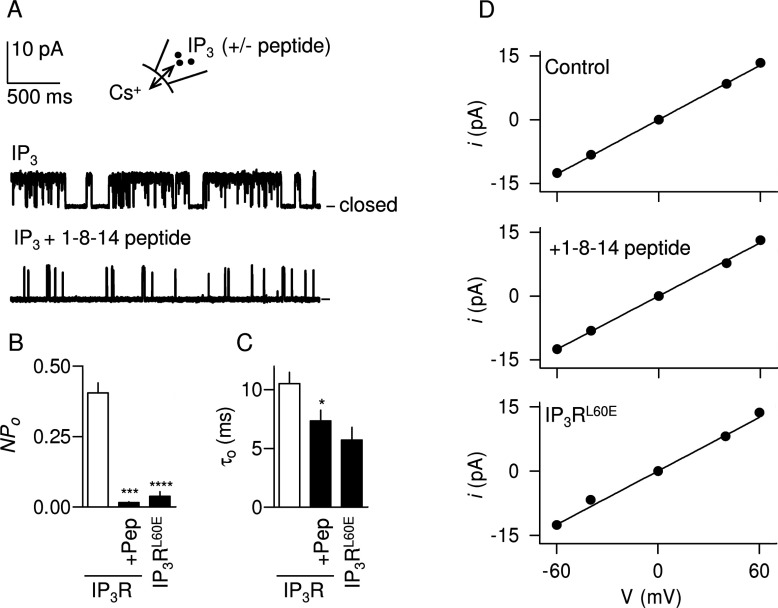
Inhibition of IP_3_R gating by 1-8-14 peptide (**A**) Typical recordings from excised nuclear patches stimulated with IP_3_ (10 μM) with and without 1-8-14 peptide (3 mM) in the pipette solution. The holding potential was +40 mV. The closed state is shown. (**B** and **C**) *NP*_o_ (**B**) and τ_o_ (**C**) for IP_3_R stimulated with IP_3_ alone or with 1-8-14 peptide (3 mM, +Pep). Results for IP_3_R^L60E^ are also shown. *P<0.05, ***P<0.001 and ****P<0.0001 relative to native IP_3_R without peptide. (**D**) Single-channel current (*i*)–voltage (V) relationships for the three stimulation conditions. Results in (**B**)–(**D**) are means±S.E.M. (*n*≥3).

In patch-clamp recordings from the nuclear envelope of DT40 cells expressing rat IP_3_R1, a maximally effective concentration of IP_3_ stimulated IP_3_R activity and this was massively attenuated by the 1-8-14 peptide (3 mM) (Figures 3A and 3B). Our results are consistent with the peptide causing a 50% decrease in the mean channel open time (τ_o_) (Figure 3C). However, the overall channel activity (*NP*_o_) was so low under these conditions that we cannot reliably estimate the number of active IP_3_Rs (*N*) within each patch. We cannot therefore entirely eliminate the possibility that each patch fortuitously included several IP_3_Rs and that their clustering caused τ_o_ to fall from ~10 ms to ~5 ms as we reported previously [40]. An effect on τ_o_ would be unusual because most regulators of IP_3_Rs affect the duration of closed states (τ_c_) [6,40]. The effect of the peptide on τ_o_ is not, however, sufficient to account for the ~10-fold decrease in *NP*_o_ (Figure 3A), suggesting that the 1-8-14 peptide must also affect the rate of channel opening (i.e. τ_c_). Because it was impossible to determine the number of active IP_3_Rs in the presence of 1-8-14 peptide (see above), we could not reliably determine τ_c_. The single-channel conductance (γ_Cs_) was unaffected by 1-8-14 peptide: it was 214±6 pS (*n*=3) and 209±6 pS (*n*=3) for control and peptide-treated IP_3_Rs respectively (Figure 3D).

These results establish that a peptide derived from an endogenous 1-8-14 motif within the SD of the IP_3_R is similar to MLCK peptide in causing substantial and reversible inhibition of IP_3_Rs that is independent of CaM. This conclusion is consistent with our suggestion that MLCK peptide inhibits IP_3_Rs by mimicking an endogenous 1-8-14 motif, and so perhaps ‘unzipping’ an interdomain interaction [43] that is essential for activation of IP_3_Rs.

### 1-8-14 peptide uncouples IP_3_ binding from activation of IP_3_Rs

Removal of the SD increases the affinity of both full-length IP_3_Rs and the NT for IP_3_ [33]. We [33] have suggested that this reflects the use of binding energy to drive conformational rearrangement of SD-IBC interfaces during the initial steps of IP_3_R activation [5,44].

**Figure 4:**
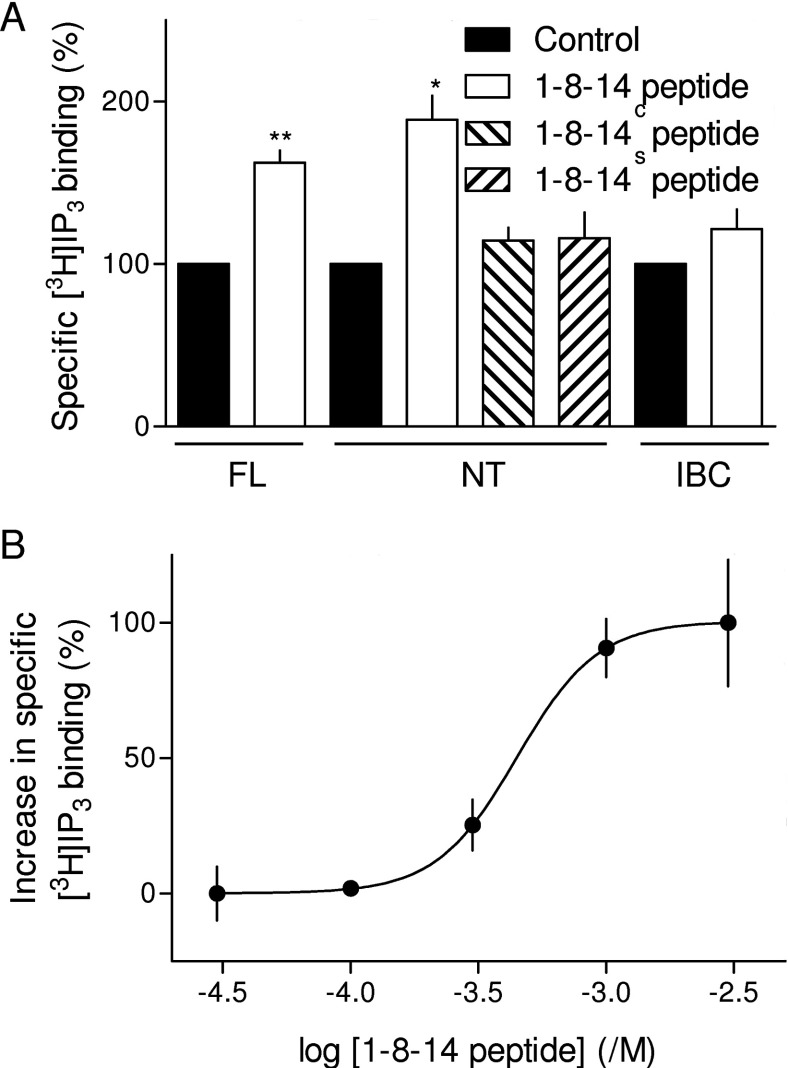
1-8-14 peptide directly stimulates IP_3_ binding to the NT of IP_3_R (**A**) Specific equilibrium binding of [^3^H]IP_3_ (1.5 nM) to membranes from rat cerebellum (full-length IP_3_R, FL) or to isolated NT or IBC, alone or in the presence of 3 mM of the indicated peptide. *P<0.05 and **P<0.01 relative to control; comparisons were performed on the raw data. (**B**) Concentration-dependent effects of 1-8-14 peptide on specific [^3^H]IP_3_ binding to NT in CLM with 220 nM free Ca^2+^ concentration, plotted as the increase in specific [^3^H]IP_3_ binding as a percentage of that evoked by the maximal concentration of peptide. Results are means±S.E.M. (*n*≥3).

1-8-14 peptide (3 mM) increased specific binding of [^3^H]IP_3_ to full-length IP_3_R1. Similar results were obtained with the NT, but IP_3_ binding to the IBC was unaffected (Figure 4A). The latter demonstrates that 1-8-14 peptide does not interact directly with either the IP_3_-binding site or with IP_3_. Neither the mutated (1-8-14^C^) nor scrambled (1-8-14^S^) peptide had any effect on IP_3_ binding to the NT (Figure 4A). These results with IP_3_R fragments expressed in *E. coli*, which lack CaM, also further support our conclusion that the effects of 1-8-14 peptide are entirely independent of CaM.

Comparison of the effects of 1-8-14 peptide on stimulating [^3^H]IP_3_ binding to the NT (EC_50_, 615 μM; pEC_50_, 3.21±0.19) (Figure 4B) with its inhibitory effect on IP_3_-evoked Ca^2+^ release (IC_50_, 767 μM; pIC_50_, 3.1±0.25) (Figure 2C) demonstrates that each is similarly sensitive to the peptide. These results are consistent with our hypothesis that the 1-8-14 peptide disrupts an interaction between the SD and IBC that is essential for IP_3_R activation. The peptide thereby inhibits IP_3_-evoked Ca^2+^ release (Figure 2) and IP_3_R activity (Figure 3) and, by uncoupling IP_3_ binding from subsequent conformational changes, it stimulates IP_3_ binding (Figure 4). Subsequent experiments used mutagenesis of residues within the endogenous 1-8-14 motif to test this hypothesis further.

### Mutations within the endogenous 1-8-14 sequence increase IP_3_-binding affinity

If, as we suggest, the 1-8-14 peptide disrupts an essential interaction between the endogenous 1-8-14 sequence and another domain within the NT, we might expect mutation of appropriate residues in the SD to both disrupt IP_3_R activation and increase IP_3_-binding affinity. We tested the latter prediction by examining IP_3_ binding to the NT in which each of the critical (1, 8 and 14) hydrophobic/aromatic residues that are important for Ca^2+^–CaM binding to 1-8-14 motifs [45] was replaced with a charged hydrophilic residue (glutamate). The same hydrophobic residues are essential for MLCK [35] and 1-8-14 (Figure 2C) peptides to disrupt IP_3_R activation.

**Figure 5:**
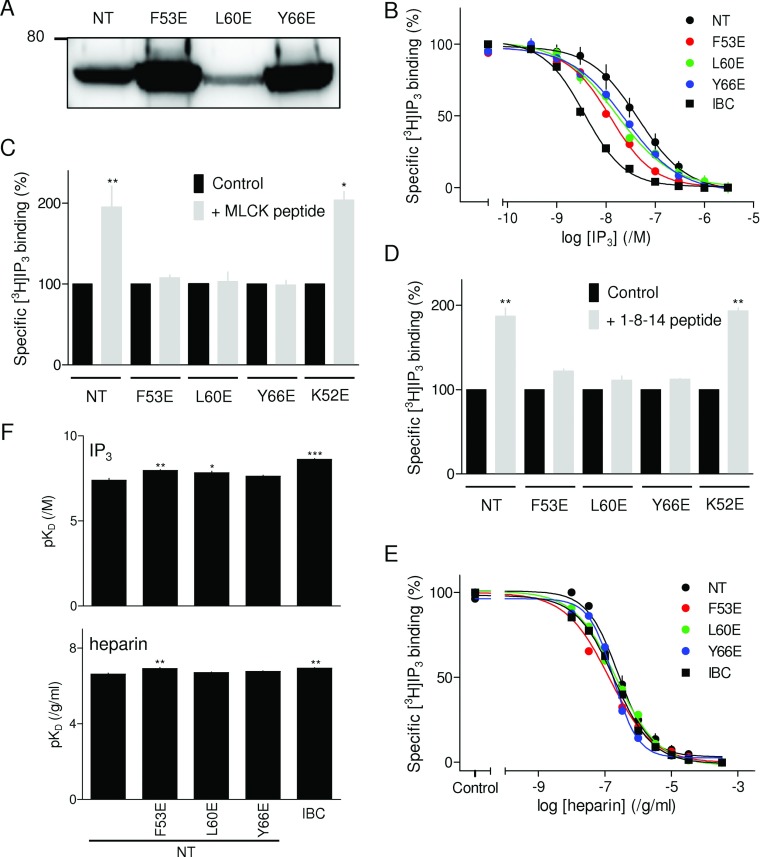
Mutations within the 1-8-14 motif mimic the effect of 1-8-14 peptide on IP_3_ binding (**A**) Western blot (typical of three independent experiments) with an anti-His_6_ antibody of lysates (5 μg of protein/lane) from bacteria expressing NT with the indicated mutations. The 80 kDa molecular-mass marker is shown. (**B**) Concentration-dependent effect of IP_3_ on specific [^3^H]IP_3_ binding to the IBC, NT and mutated NT. (**C** and **D**) Effects of MLCK peptide (**C**, 100 μM) and 1-8-14 peptide (**D**, 3 mM) on specific binding of [^3^H]IP_3_ (1.5 nM) to the NT and the indicated mutants (each expressed as a percentage of the control). (**E**) Specific binding of [^3^H]IP_3_ (1.5 nM) to the IBC, NT and mutated NT in the presence of the indicated concentrations of heparin. (**F**) Summary results from experiments similar to those in (**E**) showing the *K*_d_ for IP_3_ and heparin binding to the IBC, NT and mutated NT. Results in (**B**)–(**F**) are means±S.E.M. (*n*≥3). *P<0.05, **P<0.01 and ***P<0.001 relative to control; comparisons were performed on the raw data.

NTs of IP_3_R1 with point mutations in positions equivalent to the 1- (F53E), 8- (L60E) or 14-position (Y66E) of the endogenous 1-8-14 motif (Figure 1A) were expressed in *E. coli*. Expression levels of the NT and its mutants were not identical (Figure 5A), but they were each sufficient to allow the affinity for IP_3_ and the effects of peptides to be determined after cleavage of the His_6_ tag, but without further purification [33]. As expected, IP_3_ bound to the IBC with greater affinity (17-fold) than to the NT (Figure 5B) [33,46,47], consistent with our suggestion that, in the absence of the SD, less binding energy is diverted into conformational changes [33]. Mutation of critical residues within the endogenous 1-8-14 motif significantly increased the affinity of the NT for IP_3_ (Figure 5B and Table 1), although none was as effective as complete removal of the SD. This is consistent with our observation that neither the 1-8-14 (Figure 2) nor MLCK [35] peptide entirely inhibits IP_3_-evoked Ca^2+^ release, whereas removal of the SD totally uncouples IP_3_ binding from IP_3_R activation [48]. Although maximally effective concentrations of MLCK (100 μM) or 1-8-14 (3 mM) peptides similarly increased IP_3_ binding to the NT, neither peptide had any effect on [^3^H]IP_3_ binding to the NT with mutations in any of the critical 1-8-14 residues (Figures 5C and 5D). Mutation of a residue immediately preceding the critical 1-position of the 1-8-14 motif (K52E), which did not increase the affinity of IP_3_ for the NT (Supplementary Figure S2A at http://www.BiochemJ.org/bj/449/bj4490039add.htm), had no effect on the responses to MLCK or 1-8-14 peptides (Figures 5C and 5D) and neither did it affect IP_3_-evoked Ca^2+^ release [33] (Supplementary Figure S2B). These results establish that mutation of critical residues within the endogenous 1-8-14 motif selectively increases IP_3_-binding affinity and these effects are non-additive with those of either MLCK or 1-8-14 peptide.

**Table 1:** Binding of IP_3_ and heparin to N-terminal fragments of IP_3_R1 Equilibrium competition binding using [^3^H]IP_3_ was used to measure the p*K*_d_ of IP_3_ and heparin for the N-terminal fragments of IP_3_R1. Affinities for ligands are also shown expressed as fold increase relative to wild-type NT (i.e. *K*_d_^NT^/*K*_d_^mutant^). Results are means±S.E.M. (*n*≥3). *P<0.05, **P<0.01 and ***P<0.001 relative to NT.

Fragment	p*K*_d_, /M IP_3_ (*K*_d_, nM)	Affinity relative to NT	p*K*_d_, /g/ml heparin (*K*_d_, ng/ml)	Affinity relative to NT
NT	7.40±0.11 (40.0)	1	6.62±0.06 (239)	1
F53E	7.97±0.05** (10.8)	4	6.92±0.06** (120)	2
L60E	7.84±0.08* (14.5)	3	6.70±0.04 (200)	1.2
Y66E	7.64±0.04 (22.8)	2	6.77±0.03 (171)	1.4
IBC	8.62±0.05*** (2.4)	17	6.93±0.04** (117)	2.0

### Mutations within the 1-8-14 motif selectively increase agonist affinity

Our hypothesis is that the apparent affinity of agonists (such as IP_3_) for native IP_3_Rs is reduced because some of their binding energy is diverted into the conformational changes that activate the IP_3_R [33]. Antagonists, because they need not evoke the rearrangement of the IBC and SD that initiates IP_3_R activation, may be less affected by disruption of these interactions. We therefore examined the effects of the SD and of point mutations within the endogenous 1-8-14 sequence on binding to the NT of heparin, a competitive antagonist of IP_3_ [49]. The results demonstrate that, whereas removal of the SD increased the affinity of the NT for IP_3_ 17-fold, it caused only a 2-fold increase in the affinity for heparin. Point mutations within the endogenous 1-8-14 motif also caused larger increases in the affinity for IP_3_ than for heparin (Figures 5E and 5F, and Table 1). These results are important because they demonstrate that the effects of the SD and of mutations within the 1-8-14 sequence on ligand binding are specific for an agonist of the IP_3_R. They thereby demonstrate the importance of the 1-8-14 motif in specifically mediating activation of IP_3_Rs.

### Mutations within the endogenous 1-8-14 motif uncouple IP_3_ binding from gating of IP_3_Rs

**Figure 6:**
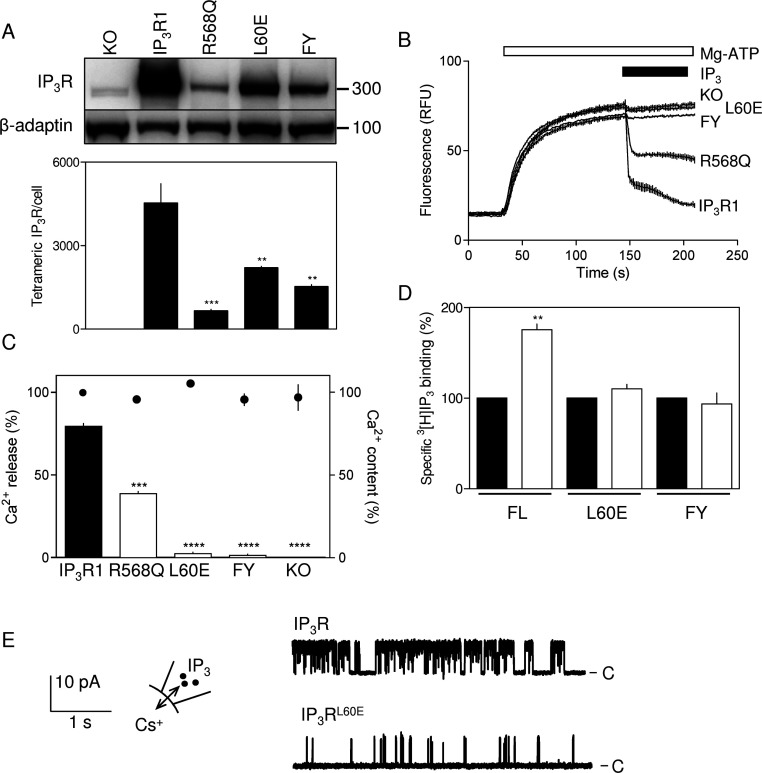
The endogenous 1-8-14 motif is essential for activation of IP_3_R (**A**) Expression of IP_3_R1 in DT40 cells stably expressing each of the indicated mutants. Each lane was loaded with 4×10^3^ cells and probed with antisera to IP_3_R1 (upper panel) or β-adaptin (lower panel). The R568Q mutant (which reduces the affinity of the IP_3_R for IP_3_) [50] is shown because it provides a control for functional assays of cells expressing IP_3_R at low density. Molecular-mass markers are shown on the right. The Western blot is typical of three independent experiments. The lower panel shows summary results (means±S.E.M., *n*=3), where IP_3_R expression was calculated from blots that included DT40-IP_3_R1 membranes in which levels of expression were established by equilibrium competition [^3^H]IP_3_ binding. (**B**) Typical responses to IP_3_ (10 μM) from DT40 cells lacking IP_3_R (KO) or expressing wild-type IP_3_R1 or IP_3_R with the indicated mutations (see the text for details). (**C**) Summary results show the Ca^2+^ content of the loaded stores (●) and the Ca^2+^ released by IP_3_ (histograms) for each of the indicated cell lines. (**D**) Specific [^3^H]IP_3_ binding (1.5 nM) to full-length IP_3_R (FL) with the indicated mutations (L60E or FY, see the text for details) in permeabilized DT40 cells alone or in the presence of 100 μM MLCK peptide. Results in (**C**) and (**D**) are means±S.E.M. (*n*≥3). (**E**) Typical records from active excised nuclear patches of DT40 cells expressing IP_3_R1 or IP_3_R1^L60E^ stimulated with IP_3_ (10 μM). The holding potential was +40 mV. C denotes the closed state. Summary data are provided in Figures 3(B)–3(D). **P<0.01, ***P<0.001 and ****P<0.0001 relative to IP_3_R1 (**A** and **C**) or control (**D**).

It proved difficult to establish stable DT40 cell lines expressing rat IP_3_R1 in which critical residues within the 1-8-14 motif were mutated, but we succeeded with two mutants (Figure 6A). The first (IP_3_R^L60E^) is mutated at the 8-position of the 1-8-14 motif and the second has mutations at both the 1- (F53E) and 14-positions (Y66E) (IP_3_R^FY^). As expected, a maximally effective concentration of IP_3_ (10 μM) failed to stimulate Ca^2+^ release from permeabilized DT40 cells lacking IP_3_R (DT40-KO cells) [37,40], but it caused release of 81±1% of the Ca^2+^ stores of DT40-IP_3_R1 cells (Figures 6B and 6C). In the cell lines expressing IP_3_R with a mutated 1-8-14 motif, there was barely detectable Ca^2+^ release that was not significantly different from that observed in DT40-KO cells (Figures 6B and 6C). ATP-dependent Ca^2+^ uptake into the ER was similar for each cell line (Figure 6C). We were concerned that the lower level of expression of mutant IP_3_R relative to wild-type (~30–50%, Figure 6A) might have contributed to the lack of detectable IP_3_-evoked Ca^2+^ release. However, in another stable DT40 cell line where the IP_3_-binding site was mutated (R568Q), causing a ~10-fold decrease in IP_3_ affinity [50], IP_3_R expression (~15% of wild-type) was less than half that of the cell lines with mutations in the 1-8-14 motif (Figure 6A). Nevertheless, IP_3_ caused a readily detectable release of Ca^2+^ from the intracellular stores of DT40-IP_3_R^R568Q^ cells (49±2% of that detected in DT40-IP_3_R1 cells) (Figures 6B and 6C). We conclude that the lack of detectable Ca^2+^ release in cells expressing IP_3_R with a mutant 1-8-14 motif is not attributable to reduced IP_3_R expression. Neither is it likely that the lack of response to IP_3_ from mutant IP_3_R reflects a more global disruption of IP_3_R structure because each of the full-length mutant IP_3_Rs bound IP_3_, although, as predicted, addition of MLCK peptide increased IP_3_ binding to only the wild-type IP_3_R (Figure 6D). Furthermore, DT40 cells expressing IP_3_R1 with a mutation in an adjacent residue (DT40-IP_3_R1^K52E^) responded normally to IP_3_ [33] (Supplementary Figure S2B). These results are consistent with the suggestion that mutations within the endogenous 1-8-14 motif mimic addition of exogenous MLCK peptide by uncoupling IP_3_ binding from the conformational changes that lead to opening of the IP_3_R pore. Single-channel analyses provide further support for this conclusion.

Yamazaki et al. [51] reported recently the functional effects of mutations within IP_3_R including some within the 1-8-14 motif (F53D and Y66A). We note, however, that some of their mutations, e.g. Y167A, which is clearly implicated in IP_3_R activation, abolished IP_3_-evoked Ca^2+^ release from microsomes without affecting Ca^2+^ signals evoked by activation of the BCR (B-cell receptor) in intact cells. This unexplained disparity casts some doubt over whether in these assays responses from intact cells faithfully report the activity of IP_3_R. In DT40 cells expressing an IP_3_R with five mutations that included Y66A (the 14-position of the 1-8-14 motif), activation of the BCR evoked a Ca^2+^ signal, suggesting that the mutant IP_3_R was functional [51]. However, in this IP_3_R, the mutant had one hydrophobic residue replaced by another and this might not radically affect the behaviour of the 1-8-14 motif. In preliminary analyses of cells expressing IP_3_Rs in which the first position of the 1-8-14 motif was mutated (F53D), Ca^2+^ signals were also observed after activation of the BCR [51]. This may reflect a limitation of the BCR-based assay (see above) or it may provide evidence for a lesser role of the 1-position in the 1-8-14 motif. We have not succeeded in establishing a DT40 cell line expressing IP_3_Rs with only this mutation, although our results do clearly show that IP_3_Rs with mutations in both the 1- and 14-positions (IP_3_R^FY^) are barely responsive to IP_3_ (Figure 6).

### Mutation of the endogenous 1-8-14 motif attenuates IP_3_R gating without affecting single-channel conductance

In keeping with the reduced expression of IP_3_R^L60E^ in DT40 cells (Figure 6A), the frequency with which functional IP_3_Rs were detected in excised nuclear patches was much lower for nuclei from paired experiments with DT40-IP_3_R1^L60E^ cells (three of 48 patches) than from DT40-IP_3_R1 cells (five of 13 patches). In parallel analyses, functional IP_3_Rs were never detected in DT40-KO cells (none of 30 patches). The single-channel conductances (γ_Cs_) of the mutant IP_3_R^L60E^ (209±8 pS) and normal IP_3_R (214±6 pS) were indistinguishable (Figure 3D), but *NP*_o_ was massively decreased in the mutant (Figures 3B and 6E). Our interpretation of the latter is, as we described in our analyses of the 1-8-14 peptide, limited by our inability, when *NP*_o_ is so low for IP_3_R1^L60E^, to estimate reliably the number of active IP_3_R within a patch. Nevertheless, it is clear that the major effect on single-channel behaviour of mutating the endogenous 1-8-14 motif of IP_3_R1 (Figures 3B–3D and 6E) and of adding 1-8-14 peptide to normal IP_3_R1 (Figure 3) is similar: both decrease *NP*_o_ without affecting γ_Cs_. These results establish that mutations in the endogenous 1-8-14 motif or addition of 1-8-14 peptide uncouple ligand binding from channel gating without compromising the behaviour of the pore.

### Conclusions: interactions between endogenous 1-8-14 and CaM-like motifs mediate activation of IP_3_Rs

**Figure 7:**
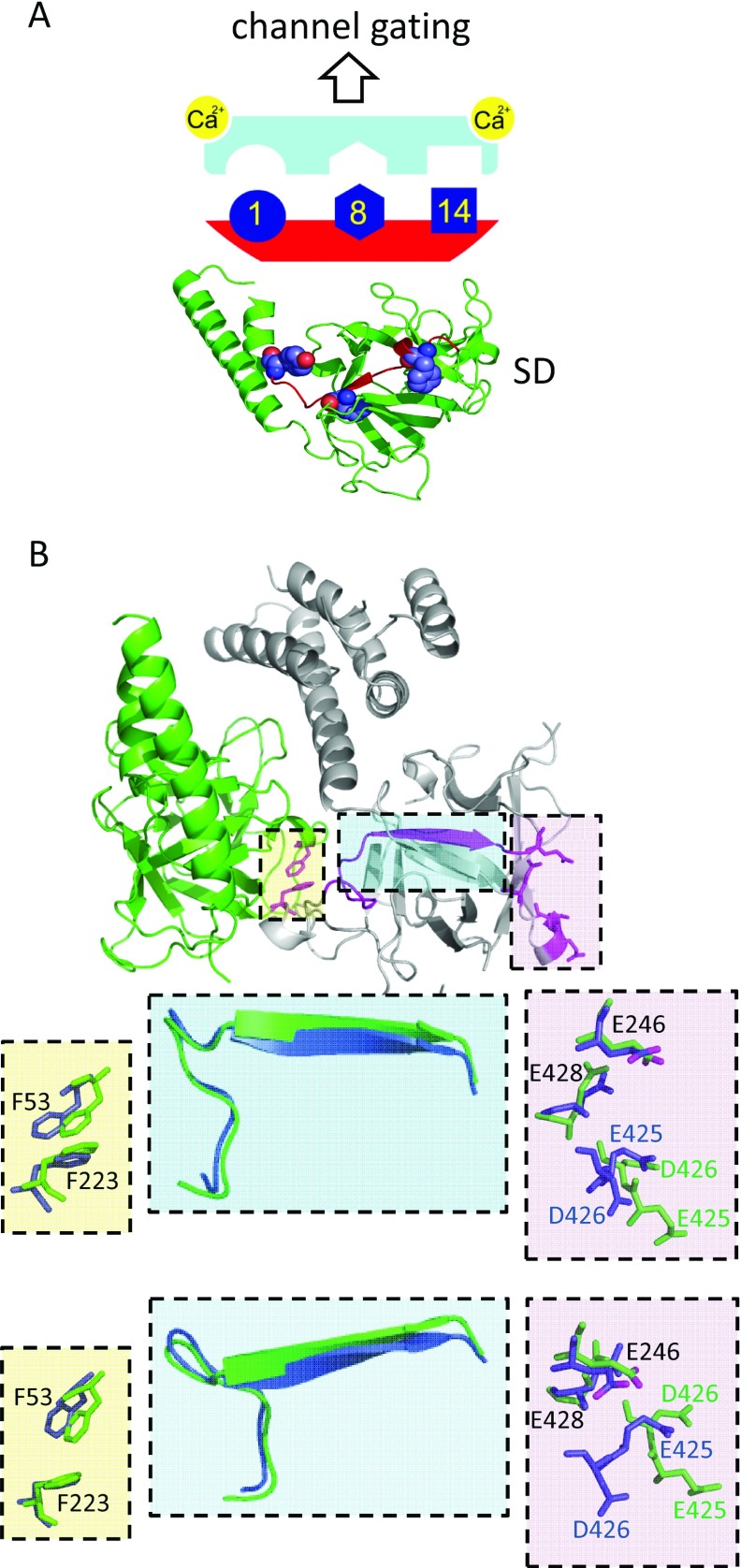
Activation of IP_3_Rs requires an endogenous 1-8-14 motif IP_3_ binding to the IBC initiates conformational changes that pass via the SD and lead, via regulation of Ca^2+^ binding to the IP_3_R, to opening of the pore [33]. (**A**) An endogenous 1-8-14 motif within the SD is essential for IP_3_R activation. We speculate (upper panel) that interaction of this CaM-binding motif (red, conserved hydrophobic residues in dark blue) with an endogenous, but presently unknown, CaM-like structure (pale blue) within the NT may link IP_3_ binding to Ca^2+^ binding. (**B**) Another possibility is that IP_3_ binding rearranges the 1-8-14 motif and so repositions a critical acidic residue (Glu^246^) that may then contribute to a Ca^2+^-binding site (Ca-1) [55]. The NT without IP_3_ bound (PDB code 3UJ0) [5] is shown with the IBC in grey and the SD in green to highlight Phe^53^ (within the 1-8-14 motif) and Phe^223^ to which it is closely apposed (yellow box), residues proposed to form the Ca-1 site (pink box) and the β-sheet that links Phe^223^ to Glu^246^ (cyan box). The expanded views (each rotated to show key movements) show the critical residues and the linking β-sheet before (green) and after IP_3_ binding (blue, PDB code 3UJ4). The carboxy oxygen atoms in Glu^246^ are shown in magenta. We speculate that separation of Phe^53^ and Phe^223^ when IP_3_ binds is associated with twisting of the linking β-sheet and movement of Glu^246^ towards three other acidic residues (Glu^425^, Asp^426^ and Glu^428^) and that they may then together form an effective Ca^2+^-binding site.

CaM [22] or related EF-hand-containing proteins [14,25], peptides that comprise 1-8-14 CaM-binding motifs [35,36] (Figures 2–4) or disruption of a conserved endogenous 1-8-14-like motif within the SD of IP_3_Rs inhibit IP_3_-evoked Ca^2+^ release (Figures 5 and 6) by massively reducing *NP*_o_ of IP_3_R (Figures 3 and 6E). We conclude that an endogenous 1-8-14 motif within the SD (Figure 1) is essential for IP_3_R activation. Where it has been examined, the inhibitory proteins or peptides are more potent when Ca^2+^ is bound to the IP_3_R [26,35] (Figure 2F). We therefore speculate that the endogenous 1-8-14 motif may interact with an unidentified domain that includes an EF-hand-like structure and that these interactions might be related to Ca^2+^ regulation of IP_3_R (Figure 7). We suggest that competing peptides (CaM-like or 1-8-14 motifs) or mutagenesis of the endogenous 1-8-14 motif inhibit IP_3_Rs by disrupting this essential interaction in a manner similar to the ‘unzipping’ of interdomain interactions in RyRs [32,43,52]. The scheme is appealing because IP_3_ regulates binding of Ca^2+^ to IP_3_Rs and thereby leads to channel gating [34,53]. The identity of this Ca^2+^-binding site is unknown. It is, however, clear that Ca^2+^ regulates IP_3_ binding to the NT only when the SD is present [42], suggesting that a Ca^2+^-binding site within the NT may be regulated by interactions between the SD and IBC. One possibility is that an endogenous EF-hand-like structure might provide the Ca^2+^-binding site and that its interaction with the 1-8-14 motif links IP_3_ and Ca^2+^ binding (Figure 7A). Bioinformatic analyses had suggested the presence of two possible EF-hand-like structures within the IBC [9,54], but neither is evident in high-resolution structures of the IBC [55] and NT [5,56]. Neither have we succeeded in identifying a complementary partner of the 1-8-14 motif. Another possibility is suggested by comparison of the structures of the NT with and without IP_3_ bound [5,56], which reveal that Phe^53^ (the first hydrophobic residue of the 1-8-14 motif) and Phe^223^ are closely apposed (~3.9 Å; 1 Å=0.1 nm), but they move apart (~5.3 Å) when IP_3_ binds (Figure 7B). A β-sheet links Phe^223^ to Glu^246^, and the movement of Phe^223^ is associated with a repositioning of an acidic residue in the β-domain of the IBC (Glu^246^). This brings Glu^246^ closer to three other acidic residues (Glu^425^, Asp^426^ and Glu^428^). The rearrangement is interesting because these four residues have been proposed to form a Ca^2+^-binding site (Ca-I) [55]. Furthermore, a peptide (residues 378–450) that includes most of these residues binds Ca^2+^, and the binding is abolished by mutation of the acidic residues [42]. A second possibility is therefore that IP_3_-evoked movement of the critical 1-8-14 motif contributes to formation of an effective Ca^2+^-binding site within the IBC by bringing a fourth acidic residue into appropriate association with three others.

We conclude that a conserved 1-8-14 motif within the SD is essential for IP_3_R activation and speculate that its interaction with either an endogenous CaM-like motif or acidic residues within the IBC may link IP_3_ and Ca^2+^ binding. Inhibition of IP_3_R by CaM and related proteins probably results from disruption of this essential interaction.

## Online data

Supplementary data
