# Supplementary material for: Activation of IP3 receptors requires an endogenous 1-8-14 calmodulin-binding motif
Source: Biochem J. 2012 Dec 7;449(Pt 1):39–49. doi: 10.1042/BJ20121034 (PMC3685217; doi:10.1042/BJ20121034)
Supplement: Supplementary data [file bj4490039add.pdf]

Biochem. J. (2013) **449**, 39–49 (Printed in Great Britain) doi:10.1042/BJ20121034

SUPPLEMENTARY ONLINE DATA

Activation of IP_3 receptors requires an endogenous 1-8-14 calmodulin-binding motif

Yi SUN[1,2], Ana M. ROSSI[1], Taufiq RAHMAN and Colin W. TAYLOR[3]

Department of Pharmacology, University of Cambridge, Cambridge CB2 1PD, U.K.

			1 8 14		
MLCK peptide			RRKWQKTGHAVRAIGRL		+6
1-8-14 consensus			1XXX5XX8XXXXX14		
rat	IP_3R1	46	LNNPPKKFRDCLFKLCPMNRYSAQKQ	71	+4
rat	IP_3R2	46	LTNPPKKFRDCLFKVCPMNRYSAQKQ	71	+4
rat	IP_3R3	45	LDNPPKKFRDCLFKVCPMNRYSAQKQ	70	+4
chicken	IP_3R1	46	LNNPPKKFRDCLFKLCPMNRYSAQKQ	71	+4
chicken	IP_3R2	48	LANPPKKFRDCLFKVCPMNRYSAQKQ	73	+4
chicken	IP_3R3	45	LDNPPKKFRDCLFKVCPMNRYSAQKQ	70	+4
frog	IP_3R1	46	LNNPPKKFRDCLFRLCPMNRYSAQKQ	71	+4
frog	IP_3R2	46	LANPPKKFRDCLFKVCPMNRYSAQKQ	71	+4
frog	IP_3R3	45	LDNPPKKFRDCLFRVCPMNRYSAQKQ	70	+4
Drosophila	IP_3R	49	LSCPPKKFRDCLIKICPMNRYSAQKQ	74	+4
C. elegans	IP_3R	124	PESPPKKFRDCLFKVCPVNRYAAQKH	149	+4
rabbit	RyR1	59	PP-DLAICCFTLEQSLSV	75	-2
rabbit	RyR2	59	PP-DLSICTFVLEQSLLV	75	-2
rabbit	RvR3	59	PP-DLCVCNFVLEOSLSV	75	-2

Figure S1 A conserved 1-8-14 motif in all IP_3Rs and RyRs

Alignments (with first and last residues numbered) of the N-terminal region of rat IP_3R1–IP_3R3 (SwissProt accession numbers NP_001007236, NP_112308 and NP_037270 respectively), chicken IP_3R1–IP_3R3 (SwissProt accession numbers XP_414438, XP_001235613 and XP_418035 respectively), *Xenopus* IP_3R1–IP_3R3 (SwissProt accession numbers NP_001084015, ABP88141 and ABP88140 respectively), *Drosophila* IP_3R (SwissProt accession number NP_730942), *Caenorhabditis elegans* IP_3R (SwissProt accession number NP_001023170) and rabbit RyR1–RyR3 (SwissProt accession numbers P11716, P30957 and Q9TS33 respectively) highlighting the residues proposed to form a 1-8-14 CaM-binding motif. The consensus sequence for a 1-8-14 motif is shown in the first row, with its three critical (1, 8 and 14 hydrophobic residues) and net charge of +3 to +6. A similar 1-8-14 motif is conserved in all IP_3R, which closely resembles a type A (1-5-8-14) motif, where position 5 is also a large hydrophobic residue. The motif within IP_3Rs differs from a classic 1-8-14 consensus sequence by having a tyrosine residue at position 14. All subtypes of RyR also have a similar 1-8-14 motif within a similar position in the three-dimensional structure, although the sequence lacks the usual net positive charge of a consensus 1-8-14 motif.

[1] These authors contributed equally to this work.
[2] Present address: Wellcome Trust Sanger Institute, Wellcome Trust Genome Campus, Hinxton, Cambridge CB10 1SA, U.K.
[3] To whom correspondence should be addressed (email cwt1000@cam.ac.uk).

© 2013 The Author(s)
The author(s) has paid for this article to be freely available under the terms of the Creative Commons Attribution Licence (CC-BY) (http://creativecommons.org/licenses/by/3.0/) which permits unrestricted use, distribution and reproduction in any medium, provided the original work is properly cited.

Biochemical Journal www.biochemj.org

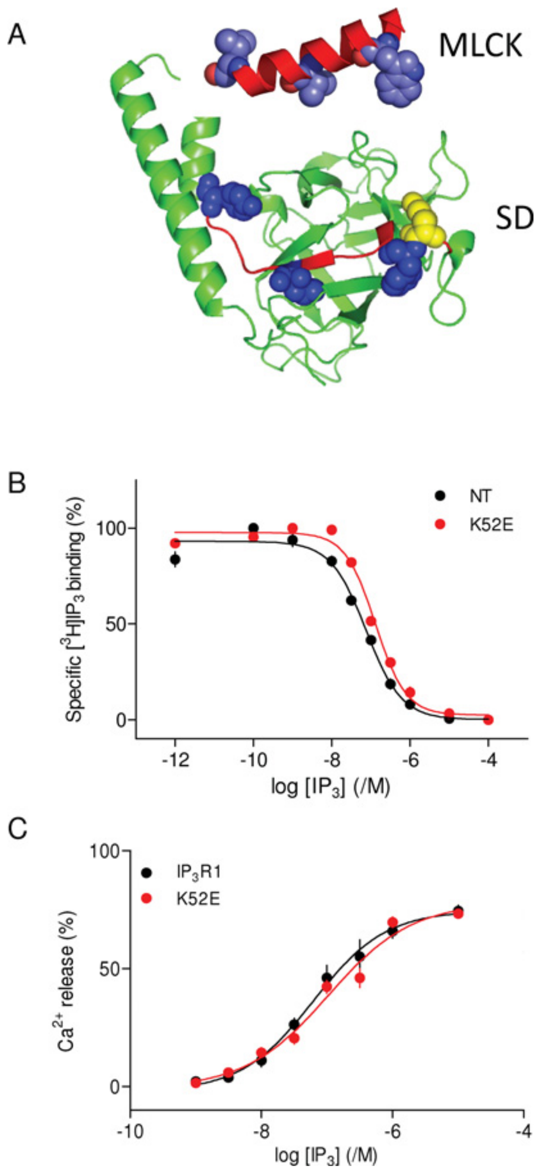

Figure S2 Mutation of a non-critical residue (K52E) within the 1-8-14 motif has no effect on IP_3 binding or IP_3-evoked Ca^{2+} release

(**A**) Structure of the SD of IP_3R1 (PDB code 1XZZ) highlighting the 1-8-14 motif (red), the critical 1-8-14 hydrophobic residues (blue) and Lys^{52} (yellow). (**B**) Equilibrium competition binding of IP_3 (with 0.75 nM $[^3H]IP_3$) to native NT and NT^{K52E}. (**C**) IP_3-evoked Ca^{2+} release from DT40-IP_3R1 and DT40-IP_3R1^{K52E} cells. Results are means ± S.E.M. ($n \geqslant 3$).

© 2013 The Author(s)
The author(s) has paid for this article to be freely available under the terms of the Creative Commons Attribution Licence (CC-BY) (http://creativecommons.org/licenses/by/3.0/) which permits unrestricted use, distribution and reproduction in any medium, provided the original work is properly cited.

Table S1 Peptides used in the present study

All peptides were synthesised by Sigma or New England Peptide. The isolelectric point (pI) is shown for each peptide calculated from http://www.innovagen.se/custom-peptide-synthesis/peptide-property-calculator/peptide-property-calculator.asp. Ac, acetyl.

Peptide	Sequence	Source	pI
MLCK	Ac-RRKWQKTGHAVRAIGRL-NH_2	Ca^{2+}–CaM-binding site of smooth muscle MLCK	14.0
1-8-14	Ac-KKFRDALFKLAPMNRY-NH_2	Fragment of IP_3R1 (residues 51–66) containing the 1-8-14 motif	11.6
1-8-14^{C}	Ac-KK**E**RDALFKLAPMNR**E**-NH_2	Inactive form of 1-8-14 peptide (mutations highlighted in bold and underlined)	10.8
1-8-14^{S}	Ac-AMRFLKYLPKRFDKNA-NH_2	Scrambled form of 1-8-14 peptide	11.6
1-8-14^{L}	Ac-LNNPPKKFRDALFKLAPMNRYSAQKQFWKA-NH_2	Longer fragment of IP_3R1 (residues 46–75) containing the 1-8-14 motif	11.7

Table S2 Primers used in the present study

Primers used for introducing mutations in the N-terminal fragment or full-length IP_3R1. The mutated bases are highlighted.

Primer	Sequence (5′→3′)
F53E Forward	GGGGACCTTAACAATCCACCCAAGAAA**GAG**AGAGACTGCCTCTT
F53E Reverse	AAGAGGCAGTCTCT**CTC**TTTCTTGGGTGGATTGTTAAGGTCCCC
L60E Forward	GAAATTCAGAGACTGCCTCTTTAAG**GAG**TGTCCTATGAATCGATATTCTGCA
L60E Reverse	TGCAGAATATCGATTCATAGGACA**CTC**CTTAAAGAGGCAGTCTCTGAATTTC
Y66E Forward	CTCTTTAAGCTATGTCCTATGAATCGA**GAG**TCTGCACAGAAGCAG
Y66E Reverse	CTGCTTCTGTGCAGA**CTC**TCGATTCATAGGACATAGCTTAAAGAG
K52E Forward	AACAATCCACCCAAG**GAA**TTCAGAGACTGCCTC
K52E Reverse	GAGGCAGTCTCTGAA**TTC**CTTGGGTGGATTGTT

Received 26 June 2012/13 September 2012; accepted 26 September 2012
Published as BJ Immediate Publication 26 September 2012, doi:10.1042/BJ20121034

© 2013 The Author(s)
The author(s) has paid for this article to be freely available under the terms of the Creative Commons Attribution Licence (CC-BY) (http://creativecommons.org/licenses/by/3.0/) which permits unrestricted use, distribution and reproduction in any medium, provided the original work is properly cited.
